# Effects of Bedaquiline Combined with Fluoroquinolone and/or Clofazimine on QT Interval in Patients with Multidrug-Resistant Tuberculosis: a Retrospective Study

**DOI:** 10.1128/spectrum.01048-23

**Published:** 2023-06-13

**Authors:** Rong Li, Jin-Bao Ma, Hong Yang, Han Yang, Xin-Jun Yang, Yan-Qin Wu, Fei Ren

**Affiliations:** a Department of Drug-resistance tuberculosis, Xi’an Chest Hospital, Xi’an, China; b Medical Transformation Center of Xi’an Chest Hospital, Xi’an, China; Johns Hopkins University School of Medicine

**Keywords:** tuberculosis, pulmonary, multidrug resistance, electrocardiography, drug toxicity, bedaquiline

## Abstract

With the application of bedaquiline (Bdq), the success rate of multidrug-resistant tuberculosis (MDR-TB) treatment has been significantly improved; however, the cardiac safety of the patients during treatment cannot be ignored. Hence, this study compared the effects of bedaquiline alone and bedaquiline combined with fluoroquinolones (FQs) and/or clofazimine (CFZ) on the QT interval. This single-center retrospective cohort study analyzed the clinical data of MDR-TB patients treated with bedaquiline for 24 weeks from January 2020 to May 2021 in Xi’an Chest Hospital and compared the changes in QTcF between the two groups. Eighty-five patients were included in the study and grouped by types of anti-TB drugs affecting the QT interval they used. Group A included bedaquiline (*n* = 33), and group B included bedaquiline in combination with fluoroquinolones and/or clofazimine (*n* = 52). Out of patients with available corrected QT interval by Fridericia’s formula (QTcF) data, 2.4% (2/85) experienced a postbaseline QTcF of ≥500 ms, and 24.7% (21/85) had at least one change of QTcF of ≥60 ms from baseline. In group A, 9.1% (3/33) had at least one ΔQTcF of >60 ms, as did 34.6% (18/52) of group B. Multivariate Cox regression analysis showed that the adjusted risk of QT prolongation was 4.82 times higher in group B (95% confidence interval [CI], 1.406 to 16.488). Bedaquiline combined with other anti-TB drugs affecting QT interval significantly increased the incidence of grade 3 or 4 QT prolongation; however, no serious ventricular arrhythmia and permanent drug withdrawal occurred. The use of bedaquiline combined with fluoroquinolone and/or clofazimine is an independent risk factor affecting QT interval.

**IMPORTANCE** Tuberculosis (TB) is a chronic infectious disease caused by Mycobacterium tuberculosis. The emergence of MDR-TB is caused by an organism that is resistant to at least isoniazid and rifampin and is currently considered the major challenge for the global control of TB. Bedaquiline is the first new TB drug in 50 years with a unique mechanism of action, strong anti-M. tuberculosis activity. Yet unexplained excess deaths in the bedaquiline arms have been found in some phase II clinical trials; thus, the FDA has issued a “boxed warning.” However, the cardiac safety of the patients during treatment cannot be ignored. Accordingly, further investigations are needed to establish whether bedaquiline combined with clofazimine, fluoroquinolones, or anti-TB drugs affecting the QT interval in a long-course or short-course treatment increases the risk of QT prolongation.

## INTRODUCTION

Tuberculosis (TB) is a chronic infectious disease caused by Mycobacterium tuberculosis. The emergence of multidrug-resistant TB (MDR-TB) is caused by an organism that is resistant to at least isoniazid and rifampin and is currently considered the major challenge for the global control of TB. Thus far, MDR-TB has been associated with poor therapeutic effect, high treatment failure rate, long treatment duration, expensive therapeutic regimen, and high incidence of adverse events (1–3). In 2021, TB deaths rose for the first time in more than a decade due to the COVID-19 pandemic, and the number of patients receiving treatment for DR-TB decreased by 15% from 2019. However, the average treatment success rate for MDR-TB and rifampin-resistant tuberculosis (RR-TB) is 57% compared with 39% for extensively drug-resistant tuberculosis (XDR-TB) (4). Therefore, new effective and safe drugs and highly effective treatment regimens are urgently needed to improve the treatment outcomes of MDR-TB and XDR-TB and prevent further drug resistance.

Bedaquiline (Bdq) is the first new TB drug in 50 years with a unique mechanism of action, strong anti-M. tuberculosis activity, and high antibacterial activity against sensitive and multidrug-resistant strains (3). It helps improve the early sputum smear/culture conversion rate, shorten the treatment duration, and enhance the therapeutic effect (5). WHO recommends bedaquiline over amikacin for the short-course treatment regimen for MDR-TB in order to avoid the ototoxicity of amikacin. Bedaquiline, combined with other group A and B drugs (specified in the groupings of anti-TB drugs of WHO guidelines), is also recommended for the long-course treatment regimen for MDR-TB. The first bedaquiline implementation study conducted in China based on the New Drug Introduction and Protection Program (NDIP) also confirmed that bedaquiline combined with background therapy could achieve a good therapeutic effect mostly with mild side effects (6). Yet unexplained excess deaths in the bedaquiline arms have been found in some phase II clinical trials (7); thus, the FDA has issued a “boxed warning” (7). Accordingly, further investigations are needed to establish whether bedaquiline combined with clofazimine, fluoroquinolones, or anti-TB drugs affecting the QT interval in a long-course or short-course treatment increases the risk of QT prolongation.

The aim of this study was to investigate the safety of bedaquiline combined with other anti-TB drugs affecting the QT interval in Chinese patients with MDR-TB.

## RESULTS

### Relationship between drug type and grade 3 or 4 QT prolongation.

**FIG 1:**
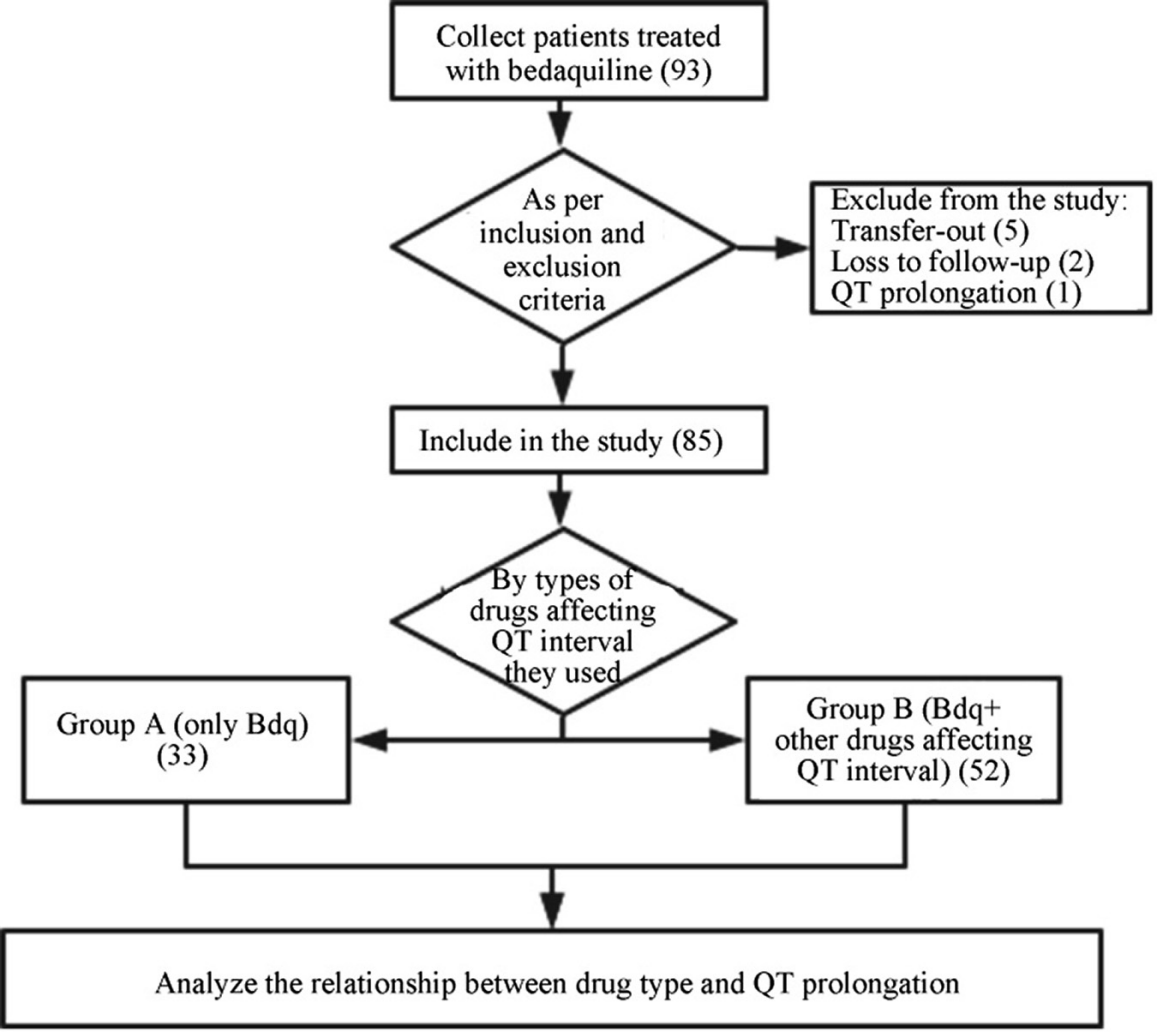
Study flowchart.

Among a total of 92 patients who were treated with bedaquiline, 5 patients were not evaluable for therapeutic effect due to transfer to other medical institutions, 2 patients were lost to follow-up, and 1 patient had QT prolongation before the use of anti-TB drugs, and they were excluded from the study. Finally, 85 patients were included in the study. The mean age of the study population was (35.96 ± 13.09) years, 37.6% (32/85) were female, and 60% (51/85) were treatment naive. The proportion of fluoroquinolone resistance and pyrazinamide use was higher in group A (*P < *0.001). There was no significant difference in age, body mass index (BMI), QTcF, electrolytes, gender, marital status, chronic diseases, treatment naive or retreatment, alcohol use, smoking, liver function (alanine aminotransferase [ALT] and aspartate aminotransferase [AST]), creatinine, and use of anti-TB drugs (linezolid, cycloserine, ethambutol, and amikacin-capreomycin) between the two groups (all *P > *0.05). Details are shown in Fig. 1 and Table 1.

**TABLE 1:** Comparison of basic and baseline information between the two groups^*a*^

Basic information	Data for:		Statistical test value	*P*
	Group A (*n* = 33)	Group B (*n* = 52)		
Age (mean ± SD [yrs])	35.82 ± 14.22	36.06 ± 12.47	*t* = −0.08	0.334
BMI (mean ± SD [kg/m^2^])	20.44 ± 2.54	20.37 ± 3.30	*t* = 0.10	0.935
Baseline QTcF (mean ± SD [ms])	401.09 ± 17.98	402.40 ± 19.70	*t* = −0.31	0.758
Baseline potassium (mean ± SD [mmol/L])	4.09 ± 0.36	4.10 ± 0.42	*t* = −0.13	0.894
Baseline sodium (mean ± SD [mmol/L])	142.15 ± 2.31	141.49 ± 3.09	*t* = 1.05	0.295
Baseline chloride (mean ± SD [mmol/L])	103.65 ± 3.03	103.20 ± 3.59	*t* = 0.59	0.483
Baseline calcium (mean ± SD [mmol/L])	2.29 ± 0.11	2.29 ± 0.14	*t* = −0.27	0.792
Baseline AST (mean ± SD [U/L])	23.06 ± 4.77	24.29 ± 12.31	*t* = −0.95	0.345
Baseline ALT(mean ± SD [U/L])	23.02 ± 7.43	26.31 ± 18.93	*t* = −0.55	0.586
Baseline creatinine (mean ± SD [μmol/L])	56.52 ± 11.51	59.52 ± 20.11	*t* = −0.78	0.44
Baseline hemoglobin (mean ± SD [g/L])	124.61 ± 17.70	126.13 ± 17.61	*t* = −0.39	0.817
Gender (no. [%])			χ^2^ = 0.23	0.632
Male	22 (66.7)	32 (61.5）		
Female	11 (33.3）	20 (38.5）		
Marital status (no. [%])			χ^2^ = 2.70	0.100
Unmarried	16 (48.5)	16 (30.8)		
Married	17 (51.5)	36 (69.2)		
Chronic diseases (no. [%])			χ^2^ = 0.05	0.818
Yes	10 (30.3)	17 (32.7)		
No	23 (69.7)	35 (67.3)		
Treatment naive or retreatment (no. [%])			χ^2^ = 0.10	0.369
Treatment naive	22 (66.7)	29 (55.8)		
Retreatment	11 (33.3)	23 (44.2)		
FQ resistant or not (no. [%])			χ^2^ = 0.38	0.540
Yes	27 (81.8)	21 (40.4)		
No	6 (18.2)	31 (59.6)		
Alcohol use (no. [%])			χ^2^ = 0.50	0.479
Yes	13 (39.39)	24 (46.15)		
No	20 (60.61)	28 (53.85)		
Smoking (no. [%])			χ^2^ = 0.50	0.633
Yes	9 (27.27)	18 (34.61)		
No	24 (72.73)	34 (65.39)		
Linezolid (no. [%])				
Yes	33 (100.00)	52 (100.00)		
No	0	0		
Cycloserine (no. [%])				
Yes	33 (100.00)	48 (92.30)		0.154
No	0	4 (7.70)		
Pyrazinamide (no. [%])			χ^2^ = 28.18	<0.001^*b*^
Yes	29 (87.90)	15 (28.8)		
No	4 (12.10)	37 (71.2)		
Ethambutol (no. [%])				0.475
Yes	2 (6.10)	6 (11.50)		
No	31 (93.90)	46 (88.50)		
Protionamide (no. [%])			χ^2^ = 16.35	<0.001^*b*^
Yes	28 (84.80)	21 (40.40)		
No	5 (15.20)	31 (59.60)		
Amikacin or capreomycin (no. [%])			χ^2^ = 1.85	0.174
Yes	8 (24.20)	20 (38.50)		
No	25 (75.80)	32 (61.50)		

a BMI, body mass index, weight (kg)/height (m^2^); AST, aspartate aminotransferase; ALT, alanine aminotransferase; FQs, quinolones; *t*, Student’s test.

b A *P* value of <0.05 indicates a statistically significant difference.

### Incidence and outcome of grade 3 or 4 QT prolongation.

Out of patients with available corrected QT interval by Fridericia’s formula (QTcF) data, 2.4% (2/85) experienced a postbaseline QTcF of ≥500 ms, and 24.7% (21/85) had at least one change in QTcF of ≥60 ms from baseline. In group A, 9.1% (3/33) had at least one ΔQTcF of >60 ms, as well as 34.6% (18/52) of group B. No serious ventricular arrhythmia or permanent discontinuation occurred. A QTcF of >500 ms occurred in 2 patients, both in group B, at weeks 4 and 16; no QT prolongation occurred after reuse of discontinued clofazimine and bedaquiline.

### Univariate Cox regression analysis of ΔQTcF of >60 ms.

Using the presence or absence of ΔQTcF of >60 ms and the time of occurrence of ΔQTcF of >60 ms as dependent variables, albumin, hemoglobin, electrolytes (potassium, sodium, chloride, and calcium), BMI (refer to the lower limit of normal), and age (refer to previous literature [8]) were divided into 2 groups for univariate analysis; univariate Cox regression analysis showed that in group B (hazard ratio [HR], 4.39; 95% confidence interval [CI], 1.29 to 14.90), age ≥45 years (HR, 3.48; 95% CI, 1.47 to 8.20) was a risk factor, and albumin ≥40 g/L (HR, 0.32; 95% CI, 0.12 to 0.82) was a protective factor, as detailed in Table 2.

**TABLE 2:** Univariate Cox regression analysis of ΔQTcF of >60 ms

Variable	β	HR	95% CI	*P*
Drug type (group B vs group A)	1.48	4.39	(1.29–14.90)	0.018^*a*^
Gender (male vs female)	−0.11	0.90	(0.37–2.17)	0.815
Albumin ≥ 35.0 g/L (yes vs no)	−1.16	0.32	(0.12–0.82)	0.017^*a*^
Hemoglobin < 110 g/L (yes vs no)	−0.52	0.59	(0.22–1.62)	0.308
Potassium < 3.9 mmol/L (yes vs no)	−0.18	0.83	(0.34–2.06)	0.690
Sodium < 140.0 mmol/L (yes vs no)	−0.32	0.73	(0.28–1.90)	0.507
Chloride < 101.0 mmol/L (yes vs no)	0.17	1.18	(0.40–3.52)	0.762
Calcium < 2.20 mmol/L (yes vs no)	−0.64	0.53	(0.20–1.36)	0.185
BMI < 18.5 kg/m^2^ (yes vs no)	−0.29	0.75	(0.31–1.81)	0.524
Age ≥ 45 yrs (yes vs no)	1.25	3.48	(1.47–8.20)	0.004^*a*^

a A *P* value of <0.05 indicates a statistically significant difference.

### COX regression model analysis of the relationship between drug type and ΔQTcF of >60 ms.

Using drug type as the independent variable and ΔQTcF of >60 ms as the dependent variable and without adjusting for any confounding factors, the risk of QT prolongation was 4.39 times higher in group B (95% CI, 1.29 to 14.90) than in group A (the control group), with a *P* value for the trend of 0.006 < 0.01. Multivariate Cox regression analysis showed that after adjusting for the variables, the risk of grade 3 or 4 QT prolongation was 4.82 times higher in group B than group A (the control group), as shown in Table 3.

**TABLE 3:** Cox regression model analysis of the relationship between drug type and QT prolongation^*a*^

Drug type	No. of patients	Unadjusted HR (95% CI)	*P*	Adjusted HR (95% CI)	*P*
Group A	33	Ref^*c*^		Ref	
Group B	52	4.387 (1.291–14.901)	0.018^*b*^	4.815 (1.406–16.488)	0.012^*b*^

a Variables set based on univariate Cox regression screening, whether adjusted albumin is <40 and whether age is <45 years.

b A *P* value of <0.05 indicates a statistically significant difference.

c Ref, Group A for reference.

### QTcF changes over time in both groups.

**FIG 2:**
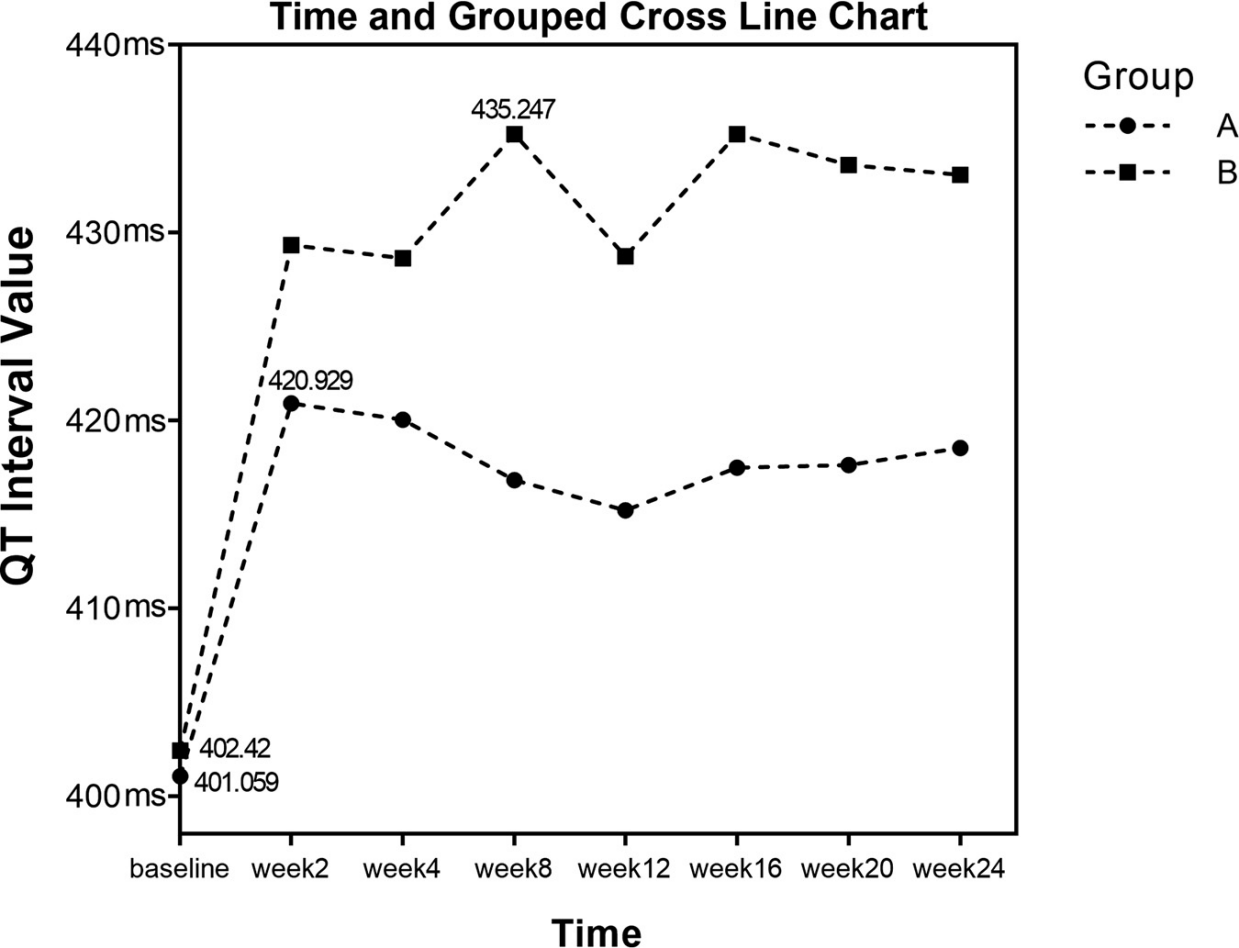
QTcF measurements of groups A and B.

**(i) QTcF change from baseline to 24 weeks in both groups.** Repeated measures analysis of variance was performed on QTcF results at different time points in the two groups, and the data did not meet Mauchly’s sphericity test results (*P < *0.001). Coefficients were adjusted by Greenhouse-Geisser correction. There was no group-by-time interaction (*F* = 2.24, *P = *0.05). The results showed a significant difference in QTcF between the two groups (*F* = 8.72, *P < *0.05), and the QTcF in group B was greater than in group A. The change in QTcF was statistically significant at different times (*F* = 18.60, *P < *0.001). The QTcF increased gradually with time after administration in both groups, reaching a maximum at week 2 after administration in group A and week 8 after administration in group B. Both groups showed a decrease at week 12, as shown in Table 4 and Fig. 2.

**TABLE 4:** Repeated measures analysis of variance results for groups A and B^*a*^

Group	Results at:								Time		Group		Group × time	
	Baseline	Wk 2	Wk 4	Wk 8	Wk 12	Wk 16	Wk 20	Wk 24	*F*	*P*	*F*	*P*	*F*	*P*
A	401.06 ± 17.96	420.93 ± 20.37	420.05 ± 20.93	416.82 ± 21.50	415.23 ± 22.76	417.49 ± 23.45	417.63 ± 22.64	418.56 ± 19.49	18.61	<0.001^*b*^	8.72	0.004^*b*^	2.24	0.05
B	402.42 ± 19.72	429.36 ± 20.03	428.66 ± 25.56	435.25 ± 24.73	428.77 ± 29.36	435.24 ± 33.77	433.61 ± 27.79	433.11 ± 30.81						

a Data are presented as mean ± standard deviation unless otherwise specified.

b A *P* value of <0.05 indicates a statistically significant difference.

### Subgroup analysis.

**FIG 3:**
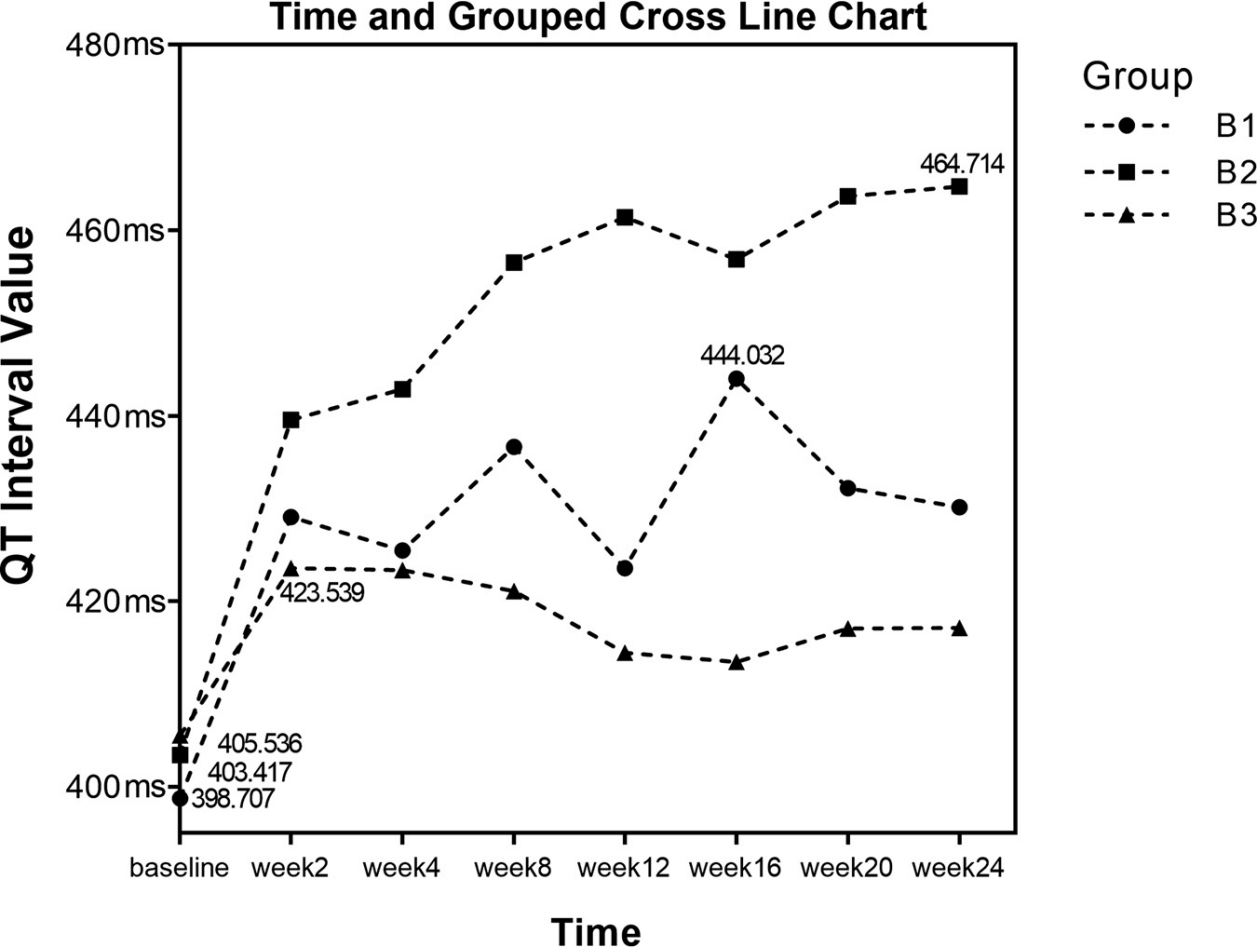
QTcF measurements of subgroups B1, B2, and B3.

Group B was divided into the following subgroups according to whether clofazimine and fluoroquinolones were used concurrently: subgroup B1 (*n* = 20), bedaquiline with clofazimine; subgroup B2 (*n* = 12), bedaquiline with fluoroquinolones and clofazimine; and subgroup B3 (*n* = 20), bedaquiline with fluoroquinolones. Repeated measures analysis of variance was performed on QTcF results at three different time points, and the data did not meet Mauchly’s sphericity test results (*P < *0.05). Coefficients were adjusted by Greenhouse-Geisser correction. There was a group-by-time interaction (*F* = 5.10, *P < *0.001), and thus, a simple effects analysis was performed. The results showed that the QTcF in subgroup B2 was greater than that in the other two subgroups; the QTcF was not the same among subgroups B1, B2, and B3, and the difference was statistically significant (*F* = 14.27, *P < *0.001). The changes in QTcF among the three subgroups were not the same at different time points, and the difference was statistically significant (*F* = 22.26, *P < *0.001). The results of the analysis of the simple effects showed that subgroups B1 and B2 had significant differences at 7 time points, and group B3 had significant differences at weeks 8, 12, 16, 20, and 24. The QTcF in subgroup B2 was greater than in subgroups B1 and B2 and continued to increase gradually, as shown in Table 5 and Fig. 3. The simple effects test at different time points in different groups is shown in Table S1 in the supplemental material.

**TABLE 5:** Repeated measures analysis of variance results for subgroups B1, B2, and B3^*a*^

Subgroup	Results at:								Time		Group		Group × time	
	Baseline	Wk 2	Wk 4	Wk 8	Wk 12	Wk 16	Wk 20	Wk 24	*F*	*P*	*F*	*P*	*F*	*P*
B1	398.71 ± 21.83	429.09 ± 21.93	425.47 ± 28.62	436.63 ± 23.60	423.53 ± 24.04	444.03 ± 37.54	432.16 ± 23.68	430.17 ± 31.48	22.26	<0.001^*b*^	14.27	<0.001^*b*^	5.10	<0.001^*b*^
B2	403.42 ± 19.41	439.52 ± 19.22	442.85 ± 26.67	456.53 ± 25.31	461.44 ± 19.51	456.91 ± 22.21	463.72 ± 20.20	464.71 ± 18.19						
B3	405.54 ± 17.99	423.54 ± 16.78	423.33 ± 18.83	421.09 ± 14.62	414.41 ± 24.39	413.46 ± 22.10	417.01 ± 20.30	417.09 ± 21.34						

a Data are presented as mean ± standard deviation unless otherwise specified.

b A *P* value of <0.05 indicates a statistically significant difference.

**FIG 4:**
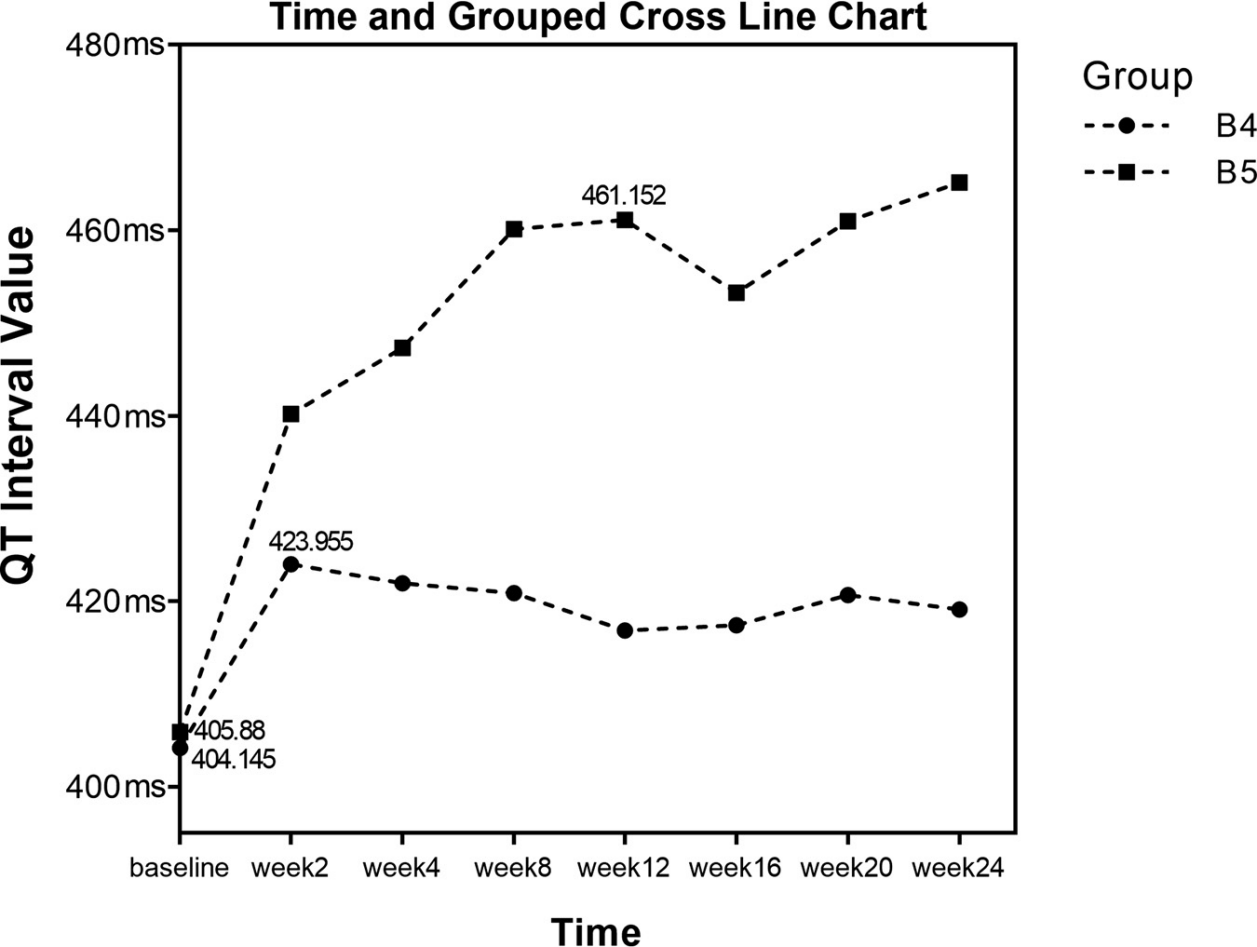
QTcF measurements of subgroups B4 and B5.

The use of fluoroquinolones in group B (*n* = 32) was divided into the following subgroups according whether levofloxacin (Lfx) and moxifloxacin were used concurrently: subgroup B4 (*n* = 21), bedaquiline with levofloxacin, and subgroup B5 (*n* = 12), bedaquiline with moxifloxacin (eight of these cases were treated with 800 mg of moxifloxacin). Repeated measures analysis of variance was performed on QTcF results at three different time points, and the data did not meet Mauchly’s sphericity test results (*P < *0.05). Coefficients were adjusted by Greenhouse-Geisser correction. There was a group-by-time interaction (*F* = 6.23, *P < *0.001), and thus, a simple effects analysis was performed. The results showed that QTcF in subgroup B5 was greater than B4; QTcF was not the same among subgroups B4 and B5, and the difference was statistically significant (*F* = 36.60, *P < *0.001); the changes in QTcF among the two subgroups were not the same at different time points, and the difference was statistically significant (*F* = 15.14, *P < *0.001). The results of the analysis of the simple effects showed that subgroups B4 and B5 had significant differences at weeks 2, 4, 8, 12, 16, 20, and 24, respectively, as shown in Table 6 and Fig. 4. A simple effects test at different time points in different groups is shown in Table S2.

**TABLE 6:** Repeated measures analysis of variance results for subgroups B4 and B5

Subgroup	Results at:								Time		Group		Group × time	
	Baseline	Wk 2	Wk 4	Wk 8	Wk 12	Wk 16	Wk 20	Wk 24	*F*	*P*	*F*	*P*	*F*	*P*
B4	404.145 ± 4.045	423.955 ± 3.868	421.904 ± 4.498	420.867 ± 3.858	416.806 ± 5.328	417.43 ± 5.577	420.654 ± 5.183	419.129 ± 4.718	15.140	0.000	32.602	0.000	6.233	0.00
B5	405.88 ± 5.589	440.177 ± 5.345	447.346 ± 6.215	460.182 ± 5.33	461.152 ± 7.361	453.273 ± 7.705	461.007 ± 7.161	465.143 ± 6.518						

## DISCUSSION

Due to excess mortality and QT prolongation, the FDA has given bedaquiline a boxed warning. Although the incidences of QTc prolongation and torsade de pointes (TdP) are not linear, the American Heart Association still recommends that the discontinuation threshold of QTc interval should be >500 ms or an increase from baseline of >60 ms (9). The incidence of TdP is considered high when the QTc is >500 ms or increases by 60 to 70 ms in the short term, and the risk of developing TdP increases by about 5 to 7% for every 10- ms increase in the QT interval (10). Therefore, this study focused on the effects of bedaquiline combined with fluoroquinolone and/or clofazimine on the QT interval. According to the study results, the change in the QT interval in patients treated with bedaquiline-containing regimens significantly increased from week 2. In general, there was a significant increase from baseline during the 6-month intensive phase; the highest value in the Bdq group was different from other groups (Bdq plus different drugs; different numbers of combined drugs). However, overall, 24.7% (21/85) had at least one change of QTcF ≥60 ms from baseline in MDR-TB patients treated with bedaquiline combined with fluoroquinolone and/or clofazimine, which is higher than the 15.7% reported in the previous NDIP program in China. Of the patients, 2.4% (2/85) had at least one follow-up QTcF of ≥500 ms, similar to 3.1% in the previous NDIP program (6). Also, no permanent discontinuation occurred, which is consistent with previous findings (11, 12), suggesting that bedaquiline-containing MDR-TB regimens have little effect on the QT interval in MDR-TB patients and can be controlled by close monitoring and symptomatic treatment.

Meta-analysis has shown that bedaquiline, fluoroquinolones, and clofazimine are associated with a low incidence of adverse effects (AEs), leading to permanent discontinuation (13). Therefore, they are often used in the DR-TB treatment regimens, yet with different effects on the QT interval. Our results showed that the QTcF change curve was basically the same between the bedaquiline group and the bedaquiline plus fluoroquinolones group. The results of another randomized trial revealed that in the absence of clofazimine, Bdq plus fluoroquinolones increased the QTc by an average of about 11.9 to 15 ms, and the incidence of QTcF of >500 ms was ≤1%, indicating that bedaquiline plus fluoroquinolones had a lower effect on the QT interval than clofazimine (14).

The results of different groups showed that the QT prolongation was more significant for bedaquiline plus clofazimine than bedaquiline alone, which was consistent with the results of Dannemann et al. In their study, Dannemann and colleagues found a mean QTcF interval increase by 41.5 ± 8.4 ms from baseline with the cefazolin (Cfz)-containing background regimen compared with only 12.9 ± 4.1 ms in patients whose background regimen did not include clofazimine (15). This may be related to clofazimine as a potent inhibitor of hERG potassium channel signaling, which mediates repolarization of the delayed rectifier potassium current, IKr, in the cardiac action potential. Mutation or inhibition of hERG can lead to QT prolongation and potentially fatal arrhythmias, including ventricular tachycardia and torsade de pointes (16).

In the present study, we found that a significant increase in the prolongation of QT interval was observed with Bdq plus FQs and Cfz, which was consistent with the findings of Isralls et al. (17). However, the results of another study showed no difference in QT prolongation among Bdq, Bdq-Lfx, Bdq+Cfz, and Bdq+Lfx+Cfz groups, meaning QT prolongation was not related to the types of drugs affecting the QT interval (16), which is inconsistent with the results of this study and is considered to be possibly related to the use of 800 mg moxifloxacin in 12 patients in subgroup B2 in this study. Previous studies have suggested that fluoroquinolones may prolong the QT interval (18). Fluoroquinolones, with different affinities for binding to the delayed rectifier potassium current, can affect ventricular repolarization (19), while moxifloxacin is more likely to cause QT prolongation than other fluoroquinolones (18). Thus, levofloxacin can be used when a variety of drugs may affect the QT interval in the regimen.

The risk factors of drug-induced QT prolongation and TdP can be summarized into the three following categories: (i) drug-related risk factors (FQs, Cfz, Bdq, delamanid [Dlm], and macrolides); (ii) individual-related risk factors (age, female, and susceptibility genes); and (iii) disease-related risk factors (preexisting cardiac diseases, hepatic and renal impairment, and hypothyroidism). The influencing factors of drug-induced QT prolongation include old age, female, acute myocardial infarction, heart failure with reduced ejection fraction, hypokalemia, hypomagnesemia, hypocalcemia, bradycardia, diuretic therapy, use of multiple drugs leading to QTc prolongation, and genetic susceptibility (10, 20). Our results showed that the risk of QT prolongation increased by 3.48 times and 4.82 times in patients >45 years old and patients taking multiple anti-TB drugs affecting the QT interval, respectively. However, QT prolongation was not related to gender, electrolyte status, and cardiac disorders, which may be due to the small sample size and the exclusion of some relevant factors when the patients were enrolled. Previous studies have shown that 10 to 20% of patients with drug-induced TdP have a genetic susceptibility, and more than 70% have at least two other risk factors, such as old age, female, and electrolyte disturbance (8). Therefore, in developing anti-TB regimens, the patient’s condition should be comprehensively considered from different aspects to determine the type, duration, and dose of drugs used leading to QT prolongation. Also, it is necessary to closely monitor electrocardiograms (ECGs), electrolytes, and other related factors.

The present study has the following limitations. First, this study had a relatively small sample size. Second, since the mean terminal elimination half-life of bedaquiline and its metabolites is about 5.5 months (21) and the half-life of Cfz after continuous administration is more than 70 days (22), the effects on the interval may persist after drug withdrawal. Also, since the QT interval data were observed for only 6 months, the QT interval during the consolidation phase should be followed by tracking. Finally, since there were many factors affecting QT prolongation and this was a retrospective study, the effects of thyroid function and other factors on QT prolongation were not considered.

Despite the above-described limitations, this study actively explored the dynamic changes of QT interval in different combinations of anti-TB drugs and provided corresponding evidence. In conclusion, QT interval prolongation occurs 4.39 times more frequently with bedaquiline plus FQs and/or Cfz than with bedaquiline alone. Therefore, factors such as age, types of drugs affecting the QT interval, and serum albumin should be considered when developing a DR-TB treatment regimen to fully assess the benefits and risks of the regimen in order to ensure the safety of patients and improve the tolerability and safety of the regimen.

## MATERIALS AND METHODS

### Subjects.

This retrospective cohort study included MDR-TB patients treated with bedaquiline in Xi’an Chest Hospital, Shaanxi Province, China, from January 2020 to May 2021. Inclusion criteria were (i) ≥18 years of age, (ii) RR- or MDR-TB confirmed by GenXpert or phenotypic drug sensitivity test, and (iii) those who received bedaquiline-containing anti-TB treatment regimens for at least 24 weeks. Exclusion criteria were the following: (i) those taking drugs affecting the QT interval other than anti-TB drugs (antiarrhythmic drugs, amiodarone and sotalol; gastrointestinal prokinetic drugs, mosapride; macrolide antibiotics, clarithromycin; psychotropic drugs, phenothiazines, etc. [23]), (ii) those with incomplete clinical information or who were lost to follow-up or transferred to other medical institutions, (iii) those with a history of cardiac disorders (arrhythmia, chronic heart failure, etc.), and (iv) with prolonged QT interval before the use of anti-TB drugs.

The study protocol was approved by the Ethics Committee of Xi’an Chest Hospital (approval no. R2022-002-01).

### Data collection.

A trained medical worker collected the following data from subjects’ medical records: demographics (gender, age, height, weight, marital status); clinical data, including drinking history, smoking history, chronic disease history (hypertension, diabetes, etc.); and electrocardiogram (ECG) (heart rate, QT interval). Among the major safety concerns of bedaquiline are alterations in the QT interval, requiring regular ECG monitoring (24). The guidelines for applying bedaquiline in China (8) require strict follow-up and ECG 2 weeks after the first dose and monthly after so that ECG results can be collected at multiple time points (baseline [within 2 weeks before taking the medication] and at 2, 4, 8, 12, 16, 20, and 24 weeks after administration). Hemoglobin, albumin, liver function, renal function, and serum electrolytes (potassium, calcium, magnesium, chloride, and sodium) were also collected at baseline.

### Definition of grade 3 or 4 QT prolongation and principles of management.

QTcF is the QT corrected by Fridericia’s formula. Grade 3 or 4 QT prolongation was defined as an absolute QT of >500 ms (QTcF > 500 ms) or a difference from baseline >60 ms (ΔQTcF > 60 ms) at any time point (25). If QTcF is >500 ms (confirmed by ECG), discontinue bedaquiline and other drugs that may cause QTcF prolongation, such as quinolones and clofazimine. After discontinuing the drug, the ECG should be rechecked every 3 days. First, add bedaquiline if QTcF is less than 450 ms, and second, add other drugs that may affect QTcF prolongation if QTcF is not in prolongation. If a QTcF of >500 ms occurs repeatedly, stop the order of clofazimine, bedaquiline, and quinolones.

### Definition of groups.

Eighty-five patients were grouped by types of anti-TB drugs affecting the QT interval they used. Group A included patients who only used bedaquiline, affecting QT interval (*n* = 33), and group B included patients who used bedaquiline in combination with fluoroquinolones and/or clofazimine (*n* = 52).

### Statistical analysis.

SPSS 23.0 software was used for statistical processing and analysis. Measurement data were analyzed by χ^2^ tests, enumeration data had normal distribution and were analyzed by *t* test, and a *P* value of <0.05 was considered statistically significant. The Cox regression model was constructed based on an adjusted set of confounding factors to analyze the relationship between drug type and QT prolongation. With group A as the control group, hazard ratio (HR) and its 95% confidence interval (CI) and *P* value were calculated. Using the presence or absence of ΔQTcF of >60 ms and the time of occurrence of ΔQTcF of >60 ms as dependent variables, significant influencing factors in univariate Cox regression were identified as the adjusted set of confounding factors (*P < *0.10). Repeated measures analysis of variance was used to compare the changes of QTcF at different time points between the two groups. If the sphericity assumption was met, the repeated measures analysis of variance was used. Otherwise, Greenhouse-Geisser correction was used instead. Simple effects analysis was performed if there was a group-by-time interaction.
