# Supplementary material for: Effects of Bedaquiline Combined with Fluoroquinolone and/or Clofazimine on QT Interval in Patients with Multidrug-Resistant Tuberculosis: a Retrospective Study
Source: Microbiol Spectr. 2023 Jun 13;11(4):e01048-23. doi: 10.1128/spectrum.01048-23 (PMC10434111; doi:10.1128/spectrum.01048-23)
Supplement: Supplemental file 1 — Supplemental material. Download spectrum.01048-23-s0001.pdf, PDF file, 0.08 MB [file spectrum.01048-23-s0001.pdf]

Attachment 1 Simple Effects Test at Different Time Points in Different Groups (Time * Group)

Time	Group	*F Value*	*P Value*
8 time points	Subgroup B1	5.89	< 0.001*
8 time points	Subgroup B2	13.57	< 0.001*
8 time points	Subgroup B3	1.54	1.670
Baseline	Three groups	0.85	0.441
Week 2	Three groups	1.83	0.185
Week 4	Three groups	1.64	0.217
Week 8	Three groups	9.00	< 0.001*
Week 12	Three groups	15.22	< 0.001*
Week 16	Three groups	6.8	0.005*
Week 20	Three groups	13.56	< 0.001*
Week 24	Three groups	11.48	< 0.001*

Note: *$P < 0.05$ indicates a statistically significant difference.

4 **Attachment 2 Simple Effects Test at Different Time Points in Different Groups (Time * Group)**

Tine	(I)Group	(J) Group	Mean Difference (I-J)	standard deviation	P	95% CI of the difference
Baseline	B4	B5	-1.735	6.899	0.803	(-15.824,12.354)
	B5	B4	1.735	6.899	0.803	(-12.354,15.824)
Week2	B4	B5	-16.222	6.598	.020 *	(-29.697,-2.746)
	B5	B4	16.222	6.598	.020 *	(2.746,29.697)
Week 4	B4	B5	-25.442	7.672	.002 *	(-41.111,-9.773)
	B5	B4	25.442	7.672	.002 *	(9.773,41.111)
Week 8	B4	B5	-39.315	6.58	.000 *	(-52.753,-25.878)
	B5	B4	39.315	6.58	.000 *	(25.878,52.753)
Week 12	B4	B5	-44.346	9.087	.000 *	(-62.904,-25.788)
	B5	B4	44.346	9.087	.000 *	(25.788,62.904)
Week 16	B4	B5	-35.842	9.512	.001 *	(-55.268,-16.416)
	B5	B4	35.842	9.512	.001 *	(16.416,55.268)
Week 20	B4	B5	-40.353	8.84	.000 *	(-58.406,-22.300)
	B5	B4	40.353	8.84	.000 *	(22.300,58.406)
Week 24	B4	B5	-46.014	8.047	.000 *	(-62.447,-29.581)
	B5	B4	46.014	8.047	.000 *	(29.581,62.447)

Note: *$P < 0.05$ indicates a statistically significant difference.
